# Tail movements by late-term fetal pitvipers resemble caudal luring: prenatal development of an ambush predatory behaviour

**DOI:** 10.1098/rsos.220218

**Published:** 2022-05-11

**Authors:** Charles F. Smith, Gordon W. Schuett

**Affiliations:** ^1^ Department of Biology, Wofford College, Spartanburg, SC 29323, USA; ^2^ Chiricahua Desert Museum, Rodeo, NM 88056, USA; ^3^ Department of Biology | Neuroscience Institute, Georgia State University, Atlanta, GA 30303, USA

**Keywords:** snakes, fetal behaviour, prenatal development, caudal luring, luring mimicry, predatory behaviour

## Abstract

With the advent of powerful imaging instruments, the prenatal behaviour of vertebrates has been discovered to be far more complex than previously believed, especially concerning humans, other mammals and birds. Surprisingly, the fetal behaviour of squamate reptiles (lizards, snakes and amphisbaenians), a group of over 11 000 extant species, are largely understudied. Using ultrasonography, 18 late-term pregnant copperhead snakes (*Agkistrodon contortrix*) from a single population were inspected for fecundity (number of fetuses). Unexpectedly, during the ultrasound procedure that involved 97 fetuses, we observed sinusoidal tail movements in 11 individuals from eight different copperhead mothers. These movements were indistinguishable from caudal luring, a mimetic ambush predatory strategy which is exhibited by newborn copperheads and other snakes. Caudal luring is initiated shortly after birth and is employed to attract susceptible vertebrate prey. Using the same ultrasound equipment and methods, we tested for this behaviour in two species of rattlesnakes (genus *Crotalus*) not known to caudal lure and none of the late-term fetuses showed any type of tail movements. Prenatal movements in humans and other vertebrates are known to be important for musculoskeletal and sensorimotor development. The fetal behaviours we describe for copperheads, and possibly other snakes, may be similarly important and influence early survival and subsequent fitness.

## 1. Introduction

Various imaging technologies clearly have opened the field of studying prenatal development and activities of vertebrates [1–6], and at present a remarkable range of behaviours is delineated for numerous taxa. Among the species that give live-birth (viviparity), prenatal behaviour is best studied in humans [1–3,7]. Behaviours documented at key developmental stages include kicking, scratching, yawning, facial expressions, and thumb-sucking to hiccupping, other vocalizations and possibly crying [1–3,7]. Prenatal vocalizations, thermoregulation and social behaviour of reptiles are recently documented [8–15]. Pre-hatch vocalizations (‘chirping’), given by many bird species [16–18] and all species of crocodilians [9,19–21], are among the most prominent examples in egg-laying (oviparous) taxa. Even some turtles are now known to vocalize prior to hatching [8–10,12]. Surprisingly, fetal behaviours are largely understudied in squamate reptiles (lizards, snakes and amphisbaenians), a group of over 11 000 extant oviparous and viviparous species [9,13,14].

Although each of the prenatal behaviours and activities previously mentioned is context-dependent (e.g. specific movements associated with musculoskeletal, motoneuron and sensorimotor development) and has different functional outcomes, they lend key insights to our overall knowledge of how developing embryos prepare for postnatal life and survival [22]. Whereas understanding normal fetal behaviour and movements in humans provides invaluable clinical perspectives for making specific diagnoses [22–24], studies of fetal behaviour in other vertebrates is important for understanding the evolutionary context for behavioural development.

Here, using ultrasonographic imaging, we show for the first time to our knowledge, that tail movements by late-term fetuses of the copperhead snake (*Agkistrodon contortrix*), a widespread viviparous pitviper from North America [25], strongly resemble caudal luring—a mimetic (ambush) predatory behaviour [26–28].

Caudal luring is a category of luring mimicry (commonly termed aggressive mimicry) often employed by newborn and juvenile snakes [26,27,29], and is most prevalent in viperids [26,29–37] but is present in several other snake lineages [38,39]. Our findings in this paper are significant for at least two main reasons. First, this is one of the few examples of prenatal behaviour in a viviparous squamate reptile (lizards and snakes). Second, sinusoidal tail movements (motor patterns) during prenatal growth have implications for the early postnatal development of caudal luring, a predatory behaviour which clearly influences survival and fitness.

## 2. Material and methods

In 2015 and 2018, a total of 18 different pregnant female copperheads (*A. contortrix*) were collected for ultrasonography (table 1). Seven females were collected from 7 July to 16 August in 2015, and 11 females were collected on 14 and 16 August 2018. The collection site was a 485 ha parcel of basalt trap rock ridge ecosystem situated 4.75 km northwest of Meriden, Connecticut [40]. In 2015, females were brought to the laboratory and provided with private enclosures, which consisted of plastic cages (61 cm L × 40 cm W × 12 cm H) supplied with paper as a floor covering and substrate heating by heat tape (8 cm wide) situated beneath and across the front end of the cage (35°C). Artificial lighting (eight 40 W fluorescent tubes) positioned 3 m above the cage was timer-controlled to simulate natural (Connecticut time) photoperiod. Water was available in glass bowls ad libitum. Because copperheads rarely eat in the latter stages of pregnancy (C.F. Smith 2009, personal observation), food (thawed mice) was not offered until after parturition. After birthing, all females (*n* = 7) brought to the laboratory were safely returned to their exact capture sites (e.g. GPS coordinates and field notes). In 2018, females were tested in the field (14 and 16 August) and released after ultrasonography testing.

**Table 1.** Ultrasound results and general data on the pregnant copperheads used in the present study. (—, no observations of luring.)

date	female ID	no. fetuses	no. lured	duration of luring (s)	parturition date	date of ultrasound	days before parturition
7 July 2015	61 688	4	—	—	21 Aug 2015	16 Aug 2015	5
7 July 2015	61 716	3	1	56	18 Aug 2015	16 Aug 2015	2
9 July 2015	61 671	6	2	39, 66	29 Aug 2015	16 Aug 2015	13
9 July 2015	61 675	5	—	—	27 Aug 2015	16 Aug 2015	11
13 Aug 2015	61 602	7	—	—	4 Sep 2015	16 Aug 2015	19
16 Aug 2015	DR1	6	2	72, 70	1 Sep 2015	16 Aug 2015	16
16 Aug 2015	DR2	3	1	85	8 Sep 2015	16 Aug 2015	23
14 Aug 2018	15 181	7	—	—	unknown	14 Aug 2018	unknown
14 Aug 2018	15 266	7	—	—	unknown	14 Aug 2018	unknown
14 Aug 2018	15 256	7	—	—	unknown	14 Aug 2018	unknown
14 Aug 2018	15 215	6	1	65	unknown	14 Aug 2018	unknown
14 Aug 2018	15 157	6	—	—	unknown	14 Aug 2018	unknown
14 Aug 2018	15 258	2	—	—	unknown	14 Aug 2018	unknown
14 Aug 2018	15 197	5	—	—	unknown	14 Aug 2018	unknown
14 Aug 2018	15 218	6	2	43, 38	unknown	14 Aug 2018	unknown
16 Aug 2018	15 236	5	1	73	unknown	16 Aug 2018	unknown
16 Aug 2018	15 165	6	—	—	unknown	16 Aug 2018	unknown
16 Aug 2018	15 245	6	1	39	unknown	16 Aug 2018	unknown

To visually access the fetuses, we scanned the pregnant subjects using a SIUI-CTS-8800 + portable ultrasound (Shantou Institute of Ultrasonic Instruments Co., Ltd., Guangdong, China) equipped with a L7L50 K-G Linear 5–12 MHz probe set at a frequency of 7.5 MHz, 60 dB in gain, and resolution depth of 3.2 cm. In 2015, parturition dates were recorded for females (*n* = 7) housed in the laboratory. Duration of scanning during the ultrasound procedure for each female was approximately 10 min; each subject was tested once. Because females captured in 2018 were released at their capture sites following the ultrasound procedure, parturition dates for these females are not known. However, all females tested in 2018 were deemed to be late-term based on the ultrasound results and time of year.

We also tested for tail movements of late-term fetuses in two other North American pitvipers, *Crotalus atrox* (*n* = 1) and *Crotalus scutulatus* (*n* = 1) from Hidalgo County, NM (Chiricahua Desert Museum, July 2019) using the same portable ultrasound equipment and procedure settings. Caudal luring has not been documented in the neonates or juveniles of either of these rattlesnake species [31,41]. We tested these rattlesnakes to determine whether the ultrasound procedure itself might be responsible for inducing tail motor patterns resembling caudal luring.

## 3. Results

**Figure 1.**
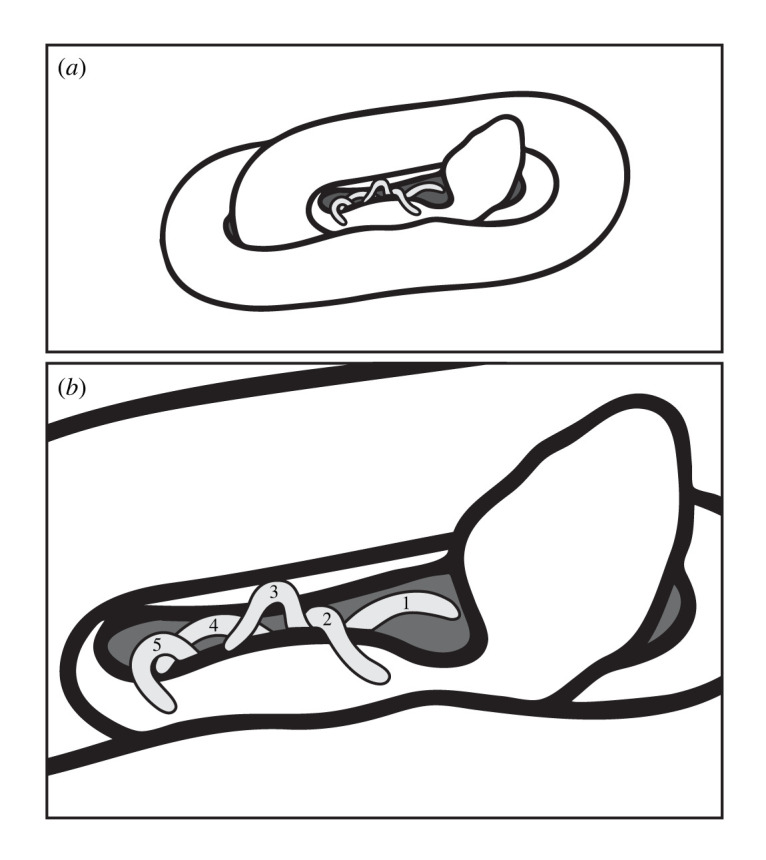
(*a*,*b*) A schematic illustrating the tail motor patterns of a single fetal copperhead. See the electronic supplementary material, videos S1 and S2. Drawing by C. F. Smith.

On 16 August 2015, the seven pregnant copperheads held in the laboratory were subjected to ultrasound testing. In total, 34 fetuses were observed via ultrasound analysis. Ultrasound observations occurred from 2–23 days prior to births (mean: 12.71 ± 2.82 s.e.). Six fetuses were recorded to produce sinusoidal tail movements, i.e. caudal luring-like motor patterns (figure 1 and table 1; see the electronic supplementary material, videos S1 and S2). The duration of tail movements was 39–85 s (*n* = 6; $\bar{x}=65\;s\pm 6.54\;s.e.$). Parturition occurred in the laboratory from 18 August to 8 September (min–max: 3–7 neonates per litter; mean litter size: $\bar{x}=4.86$, *n* = 7 females).

On 14 and 16 August 2018, 11 females were collected at the same location as in 2015. A total of 63 fetuses were observed via ultrasound as measured in the field the day they were captured. Five fetuses were recorded to produce caudal luring-like tail motor patterns (figure 1 and table 1). See the electronic supplementary material, videos S1 and S2). The duration of tail movements was 39–73 s (*n* = 5; $\bar{x}=52\;s\pm 7.26\;s.e.$). The females in this group were released at their exact capture sites following the ultrasound procedure; consequently, parturition dates for them are not know; however, based on ultrasonography, the projected litter size was min-max: 2–7 fetuses ($\bar{x}=5.72$, *n* = 11 females).

The duration of luring was not significantly different in 2015 and 2018 (*t*-test: *t*-value = 1.354, *p* = 0.209, not significant at *α* = 0.05; two-tailed, *n* = 11). Furthermore, in 2018, when using ultrasonography to project litter size, mean litter size for females was not significantly different in 2015 and 2018 (*t*-test: *t*-value = −1.216, *p* = 0.242, not significant at *α* = 0.05; two-tailed, *n* = 18).

Testing procedures of the two rattlesnake species were identical to those of the copperheads. After 10 min of scanning no tail movements of any type were observed in the 13 late-term fetuses of *C. atrox* (*n* = 1) and 11 late-term fetuses of *C. scutulatus* (*n* = 1). No other fetal behaviours were observed.

## 4. Discussion

The tail movements of late-term copperhead fetuses we describe here are compellingly indistinguishable in form and appearance to actual caudal luring, a complex ambush predatory behaviour involving mimetic resemblance to wriggling worms (sensory exploitation) in newborn, juvenile and adult copperheads (electronic supplementary material, videos S1 and S2; C.F. Smith, G.W. Schuett 2015, personal observation). Caudal luring occurs in other viperid taxa [32–37] and in several other snake lineages [31,39,41]. Because snakes are extremely sensitive to air- and substrate-borne vibrations [42–44], it is possible that handling and use of ultrasonography incited the tail movements we have described; few studies have investigated motivation or stimulus control in caudal luring [26,31,45]. Accordingly, we also tested two closely related snake species (rattlesnakes) that do not caudal lure to determine whether identical measurement procedures would produce similar results. None of the late-term fetuses in the two rattlesnake species we tested exhibited tail movements. However, owing to the small sample of control subjects, a more thorough analysis will be needed in the future to adequately address whether tail movements occur during ultrasound testing in late-term fetuses of non-luring species, such as the two we used. Nonetheless, it was not the intent of this study to conduct a rigorous statistical comparison between copperheads and other pitviper species; rather we describe for the first time, to our knowledge, a new *in utero* behaviour by a squamate reptile. Furthermore, we suggest that the tail movements by late-term fetal copperheads are potentially an essential developmental precursor to actual caudal luring, wherein the musculature and neuromotor systems are being prepared for an important postnatal activity. These tail movements may occur randomly and spontaneously, perhaps a form of fetal ‘motor babbling’ [46].

Although the duration of tail movements in the copperhead fetuses was relatively brief when compared to caudal luring in juveniles and older snakes ([29,32]; C.F. Smith 2014, personal observation), this was not unexpected. Fetal movements in vertebrates, in general, are shorter lived than the same (or similar) ones exhibited postnatally [46].

Despite the fact that our two-dimensional ultrasonographic equipment adequately and clearly documented the tail movements of late-term fetal copperheads, ultrasound and other imaging technologies have undergone significant improvement over the past few decades. Imaging accuracy, for example, is enhanced with three- and four-dimensional ultrasonography and HDlive rendering lightning systems, coupled with enhanced software and computational analysis [25,26]. We envision the next steps in the study of fetal tail movements in snakes to employ these newer technological advancements and improved methods of analysis [4,47].

From extensive work on vertebrates, especially avian reptiles (e.g. chicks), different types of fetal activity appear to be important in the ontogeny of development [46,48]. In the developing fetus, establishment of the musculoskeletal system (e.g. cartilage, joints and bone) is accomplished by mechanical load generated by muscle contractions [49,50]. Similarly, though less understood, the development of muscles follows similar steps. Results from studies of chick embryos indicate novel molecular signalling mechanisms (TAP, JAG2, NOTCH) that act downstream of muscle contraction: YAP activates JAG2 expression in muscle fibres, which subsequently influence fetal muscle progenitors by way of NOTCH [51]. Correspondingly, fetuses undergo motor neuron differentiation and sensorimotor development [46].

## 5. Conclusion

From a functional viewpoint, the abundance and diversity of fetal movements and sounds in humans and other vertebrates appear to be obligate activities for successfully transitioning to postnatal environments [47]. This also may be the case for tail movements in fetal snakes that caudal lure shortly after birth. In copperheads, and in other snakes that exhibit caudal luring, this behaviour is exhibited shortly after their first ecdysis (natal shedding), typically which occurs from 6 to 10 days after birth [52]. Although individuals of this species are born with a small bolus of yolk that supplies nourishment and energy [52], luring for and feeding on appropriately sized prey (e.g. frogs and lizards) occurs within days after their natal ecdysis. Repeated success in securing prey in early life stages typically results in rapid growth in many snake taxa, which probably promotes survival and fitness [53–55]. The present study was limited to a single viviparous species that exhibits caudal luring, yet we anticipate the same type of tail movements we documented in late-term fetal copperheads will be present in egg-laying (oviparous) taxa that exhibit caudal luring, particularly in pitvipers (e.g. *Calloselasma rhodostoma*), true vipers (e.g. *Pseudocerastes urarachnoides*), and certain species of pythons (e.g. *Morelia viridis*). It is our perspective that highly focused academic-zoo research collaborations could greatly accelerate important advancements in the study of fetal behaviour of squamate reptiles [28,56–59].

## Data Availability

Additonal material available in the electronic supplementary material [60].
